# Supplementary material for: ACMTF-R: Supervised multi-omics data integration uncovering shared and distinct outcome-associated variation
Source: PLoS One. 2026 Jan 12;21(1):e0339650. doi: 10.1371/journal.pone.0339650 (PMC12795394; doi:10.1371/journal.pone.0339650)
Supplement: Attachment — Submitted filename: REVIEWERS.docx [file pone.0339650.s003.docx]

**REVIEWERS' COMMENTS (TO EDITOR):**

The introduction of ACMTF-R effectively bridges unsupervised data exploration (via ACMTF) with supervised prediction by incorporating a regression term and tuning parameter (π). This allows for the identification of common, local, and distinct (CLD) variation linked to an outcome variable, addressing a key limitation in existing methods like ACMTF (which lacks supervision) and N-way Partial Least Squares (NPLS, which cannot handle multi-block CLD structures). The mathematical formulation is clear and well-derived, with gradients provided in supplementary materials.

The paper includes comprehensive simulations that systematically assess ACMTF-R's performance. It demonstrates the method's ability to recover small, hidden components (e.g., with norm δ=0.03) that ACMTF misses, its robustness to biologically realistic noise levels (e.g., 30-50% on X blocks), and the impact of π on balancing exploration vs. prediction. Metrics like Factor Match Score (FMS) and Tucker Congruence Coefficient are appropriately used, with clear interpretations (e.g., FMS=1 for perfect recovery).

The application to a longitudinal multi-omics dataset (human milk metabolome, HM microbiome, infant fecal microbiome) is well-chosen and yields novel findings, such as ppBMI-associated enrichments in specific genera (e.g., Bifidobacterium bifidum in high-ppBMI) and metabolites (e.g., unidentified HMOs). The method outperforms ACMTF in isolating ppBMI-exclusive components and NPLS in capturing CLD structures, providing interpretable results (e.g., time mode loadings showing stable ppBMI effects).

The provision of an open-source R package (CMTFtoolbox on CRAN/GitHub) with functions for model fitting, prediction, and selection enhances reproducibility. Default parameters and stopping criteria are justified, and computational efficiency is noted (fits in

The manuscript is well-structured, with detailed notation, methods, and supplementary materials (e.g., derivations, figures). Figures effectively illustrate simulation results and application interpretations.

**Comments and suggestions to Authors.**

----------------------------------------------------------------------------

**Abstract:**

The abstract is well organized. More importantly, it makes it easy to effectively distinguish or identify the major components of an abstract, which include the background, aims/goals, method, results, and conclusion.

----------------------------------------------------------------------------

**INTRODUCTION**

Weakness

1. Some phrases (e.g., on ACMTF's emergence and the gap between exploration/prediction) overlap closely with the abstract, which could be streamlined to avoid redundancy.
2. While citations are appropriate (e.g., 1-13 for background), they cluster early; adding a few more recent references (post-2020) on multi-omics integration or tensor methods could strengthen timeliness, especially given the field's rapid evolution.

----------------------------------------------------------------------------

**METHODS**

Weakness

1. The methods assume a shared first mode (subjects) across all blocks and y, restricting applicability to datasets with mismatched modes or higher-order tensors. While noted, no discussion of extensions (e.g., to non-shared modes) is provided, potentially limiting generalizability.
2. The tuning parameter π requires manual selection via simulations, with risks of overfitting at low values (e.g., π<0.3). Random initializations (100 per model) address local minima but increase computational burden; no automated selection (e.g., via BIC or elbow method) is proposed beyond cross-validation for component number.
3. Simulations fix most components at norm 1, with only one varying (δ), and use Gaussian noise, which may not capture real-world complexities like correlated noise or non-Gaussian distributions. Recovery issues in low-noise cases (e.g., η_X=0) are observed but not deeply analyzed (e.g., via optimization landscape). The fixed CLD structure (2 common, 3 local, 3 distinct) could be expanded to test robustness to varying structures.
4. While TCC and FMS are used effectively, their sensitivity (e.g., TCC to sign flips, addressed via absolute value) is mentioned briefly. The Hungarian algorithm for matching is appropriate but assumes known CLD types; real applications might require additional validation (e.g., against biological priors).
5. The application preprocessing (e.g., arbitrary sparsity thresholds of 75-85%, pseudo-count=1) is justified but dataset-specific; broader guidance on handling compositional data or missing values in multi-omics would strengthen the section. The intersection of subjects (reducing to 158) is necessary but could introduce bias if dropouts are non-random.

----------------------------------------------------------------------------

**RESULTS AND DISCUSSION**

This section can further be improved.

Four simulations comprehensively test ACMTF-R's performance under varying conditions (hidden component size δ, noise on X η_X, noise on y η_y). Results clearly show improved recovery (e.g., FMS, TCC >0.95) at intermediate π (0.6-0.9), robustness to biological noise (30-50%), and limits (e.g., fails at δ<0.03 or no noise). Figures (3-6) and supplements effectively visualize improvements over ACMTF.

1. Some results (e.g., higher Bifidobacterium in high-ppBMI) contradict literature without deep explanation or validation. Discussion acknowledges but could strengthen with hypotheses or sensitivity analyses.
2. Low π leads to overfitting, but results don't quantify prediction error (e.g., RMSE on hold-out set) beyond variance explained. Application focuses on π=0.9, but comparative predictive performance vs. NPLS is limited.
3. Fixed structures (e.g., 8 components, norm 1 except hidden) may not generalize; no-noise failure is noted but unexplained (e.g., no optimization diagnostics). Lacks sensitivity to varying CLD patterns or higher ranks.
4. Single dataset; CLD interpretation relies on relative λ, but all blocks contribute (issue noted in discussion). Time loadings "flat" but peaked at day 30 in HM microbiome—could explore more.
5. Dense figures; supplements referenced heavily. No direct comparison of recovered vs. true in application (as in simulations).

I would have liked to see a more detailed discussion regarding the results presented in this study and if possible the implications of the results. In other words, the results are not well discussed.

The authors should carefully correct all typos in the current version of the work
